# Supplementary material for: Identification of Parkinson’s disease PACE subtypes and repurposing treatments through integrative analyses of multimodal data
Source: NPJ Digit Med. 2024 Jul 9;7:184. doi: 10.1038/s41746-024-01175-9 (PMC11233682; doi:10.1038/s41746-024-01175-9)
Supplement: Supplementary file 1 — Supplementary Information [file 41746_2024_1175_MOESM1_ESM.pdf]

Supplement to: Identification of Parkinson's disease PACE subtypes and repurposing treatments through integrative analyses of multimodal data

Supplementary Note 1. Determination of optimal cluster number

A. Subtype identification in the PPMI (development) cohort

Using learned representation vectors of participants in the PPMI cohort, dendrogram showed that the 3-cluster model is the optimal fit of the agglomerative hierarchical clustering model (see Supplementary Figure 1). In addition, out of 18 indices in 'NbClust', 8 suggested 3 clusters, 1 suggested 1 cluster, 4 suggested 2 clusters, 3suggested 4 clusters, and 2 suggested >7 clusters. In conclusion, by considering both dendrogram and the indices, the optimal cluster number was 3.

B. Subtype identification in the PDBP (validation) cohort

In the PDBP validation cohort, dendrogram also showed that the 3-cluster model is the optimal fit of the agglomerative hierarchical clustering model (see Supplementary Figure 2). In addition, out of 18 indices in 'NbClust', 5 suggested 3 clusters, 1 suggested 1 cluster, 5 suggested 2 clusters, and 5 suggested 4 clusters, and 1 suggested 8 clusters. In conclusion, by considering both dendrogram and the indices, the optimal cluster number was 3.

Supplementary Figures

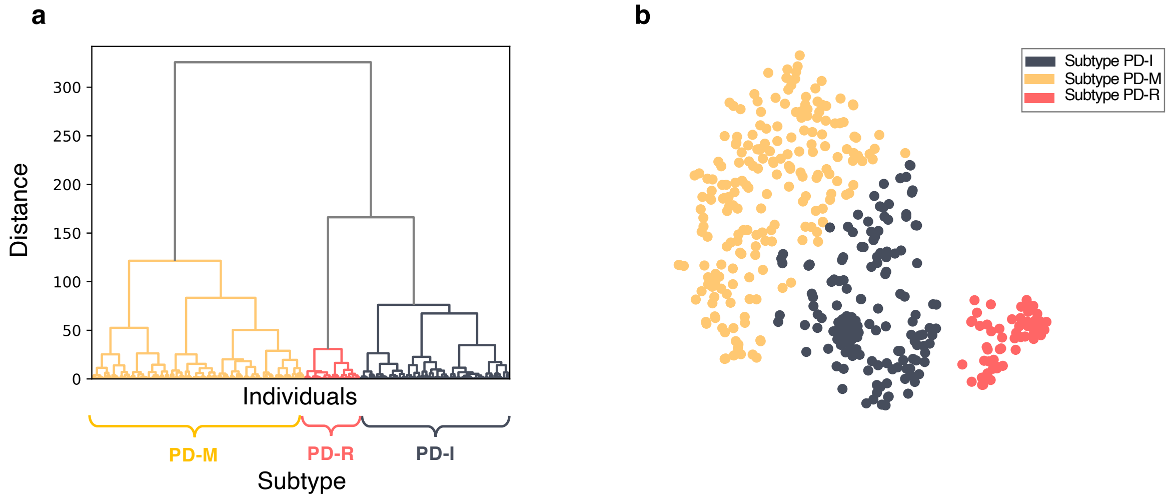

Supplementary Figure 1. Performance of hierarchical clustering in the PPMI cohort. a. Dendrogram of hierarchical clustering shows clear three cluster structure of PDs in PPMI data. **b.** t-Stochastic Neighbor Embedding (t-SNE) visualization of shows clear three cluster structure of PDs in PPMI data.

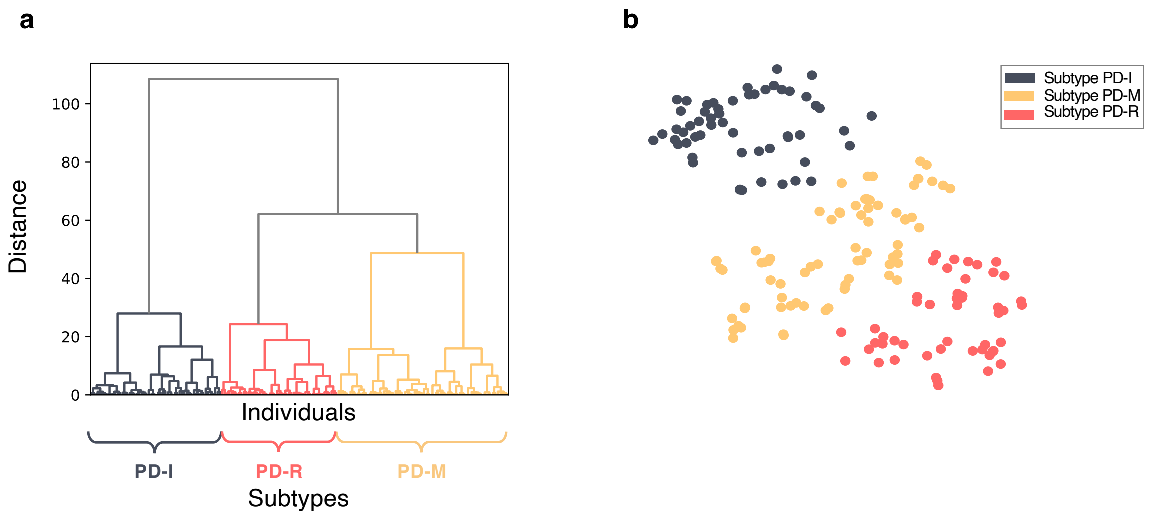

Supplementary Figure 2. Performance of hierarchical clustering in the PDBP cohort. a. Dendrogram of hierarchical clustering shows clear three cluster structure of PDs in the PDBP data. **b.** t-SNE visualization of shows clear three cluster structure of PDs in the PDBP data.

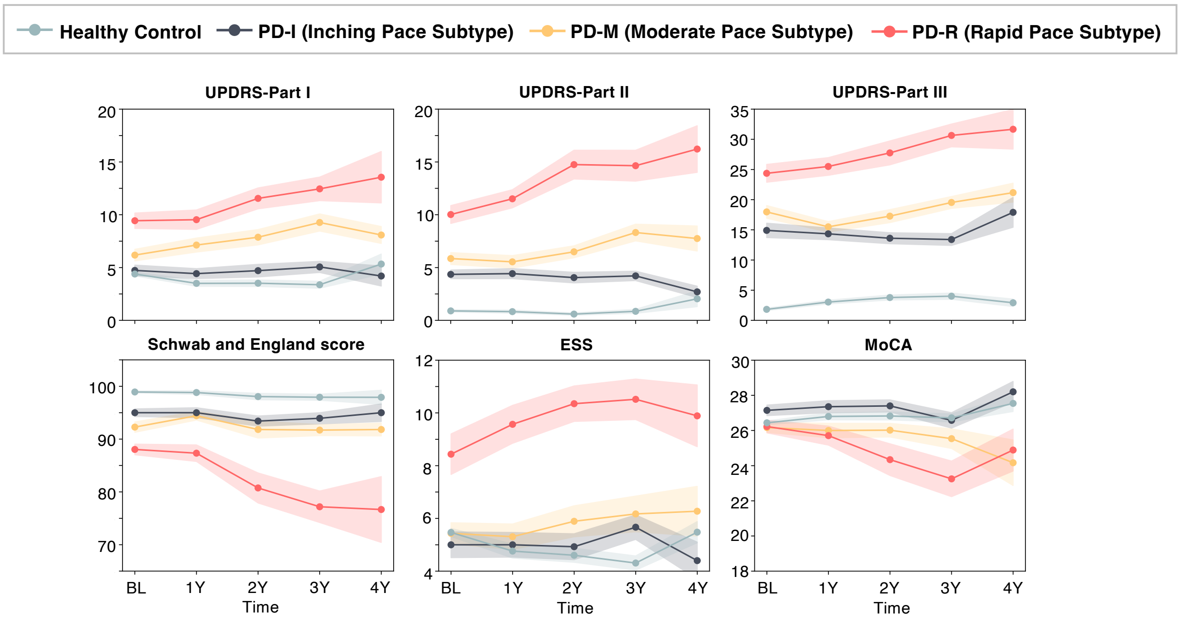

Supplementary Figure 3. Averaged symptom progression trajectories by PD subtypes in the PDBP cohort. PD symptom progression profiles of these re-identified subtypes closely mirrored those uncovered in our primary analysis within the PPMI cohort.

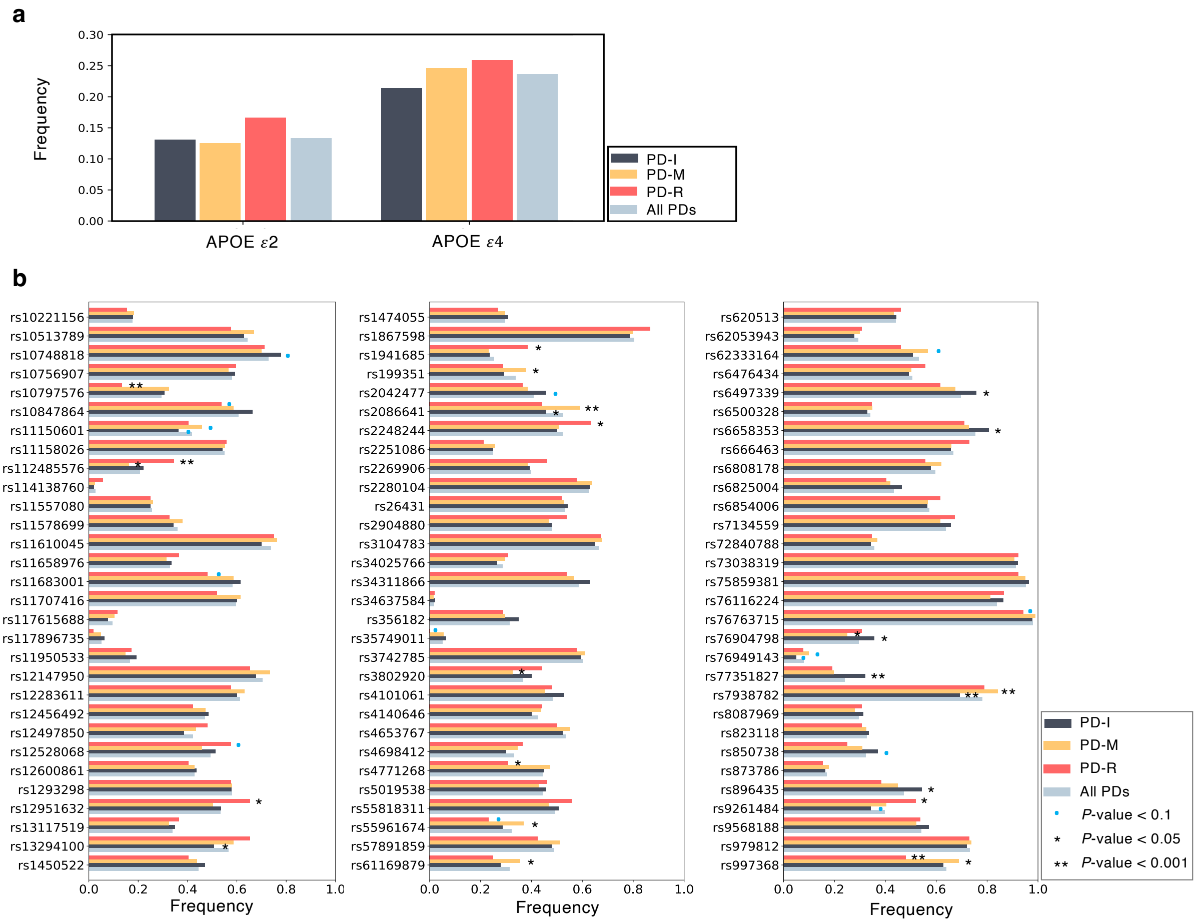

Supplementary Figure 4. Results of genetic analysis across subtypes. Enrichment analysis didn't find difference in APOE ε2 and ε4 alleles, GBA and LRRK2 variants among identified PD subtypes (**a**). Signals in 90 PD-related SNPs were found to be associated with the identified PD subtypes (**b**).

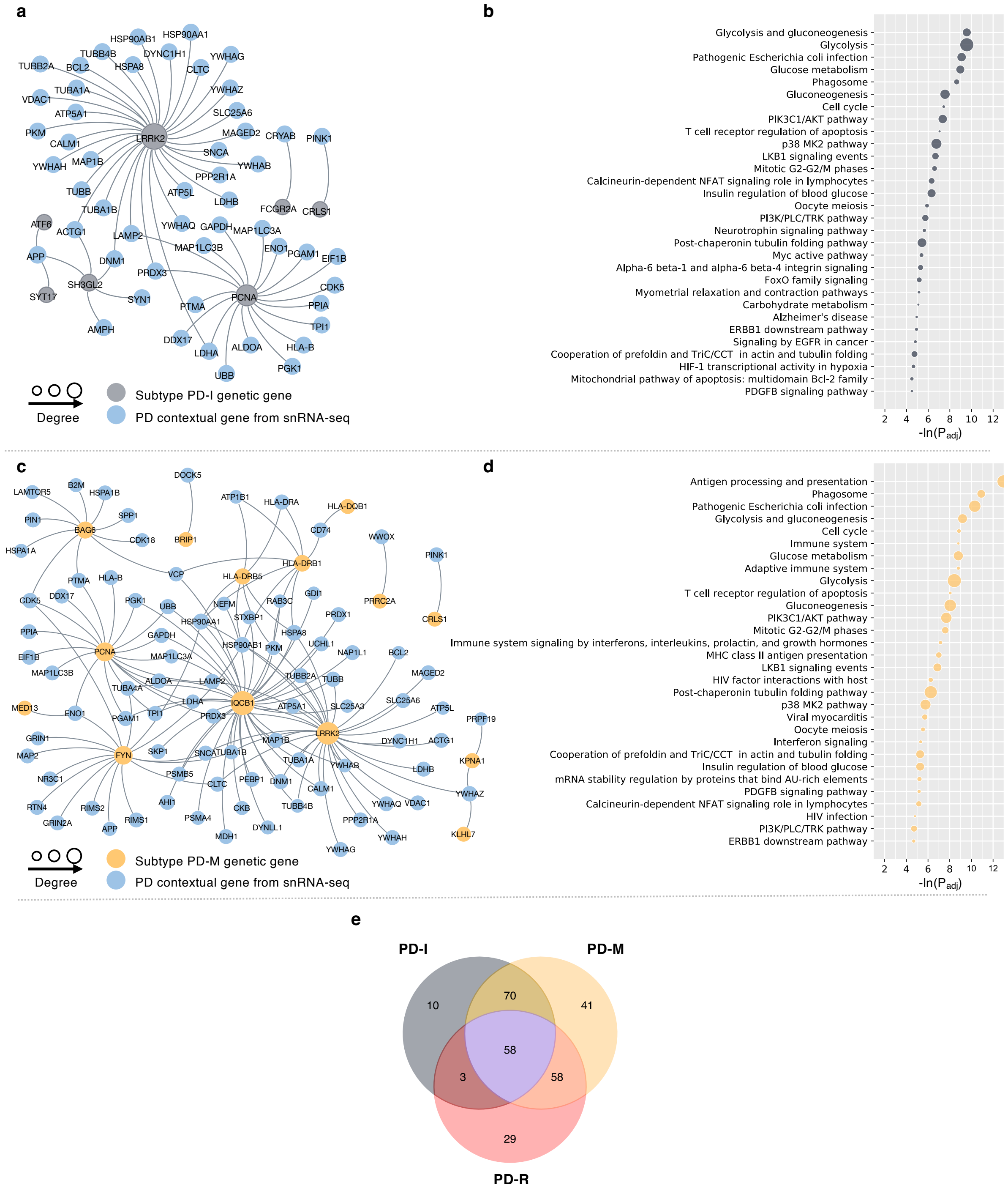

Supplementary Figure 5. Genetic molecular modules of the subtypes. **a.** and **b.** Genetic molecular module and pathways enriched based on genetic molecular module of the PD-I subtype. **c.** and **d.** Genetic molecular module and pathways enriched based on genetic molecular module of PD-M. **e.** Venn plot showing overlaps of enriched pathways among the three subtypes.

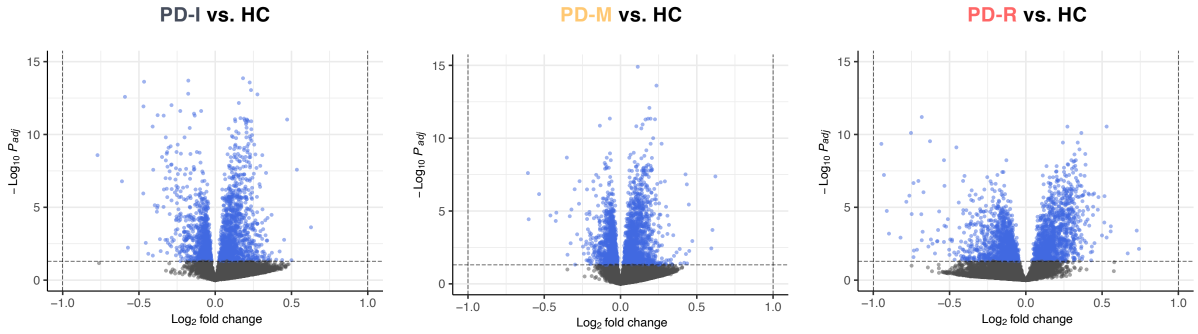

Supplementary Figure 6. Volcano plots for differential gene expression analysis. Genes with adjusted *P* value (i.e., *Q* value) < 0.05 were considered as differentially expressed genes (DEGs) in each subtype (subtype vs. healthy controls [HCs]), which were further fed to the GPSnet algorithm for identifying gene modules of each of the identified PD subtypes.

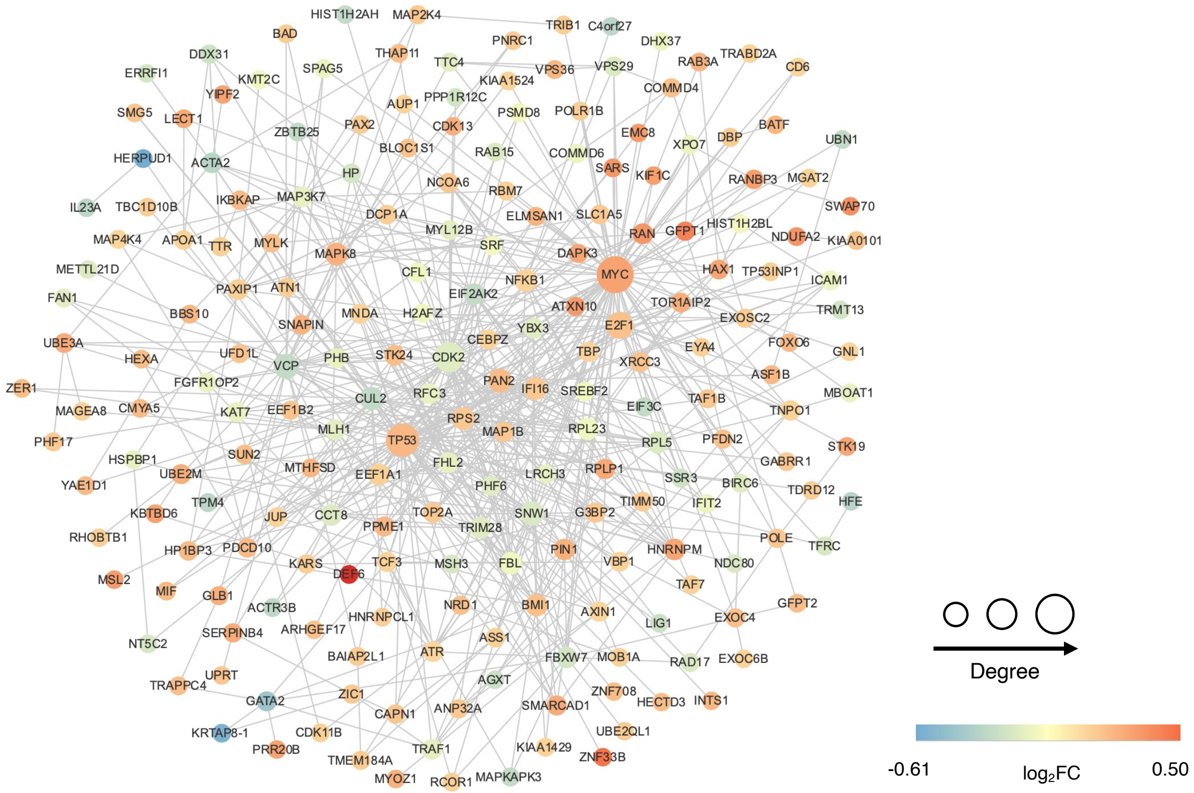

Supplementary Figure 7. Transcriptomic molecular module of the PD-I subtype identified based on gene expression profiles with network-based method, GPSnet. Color indicates log fold change in gene expression, PD-I vs. healthy control. Size of a gene indicates degree (number of connected genes) of the gene in the protein-protein interaction network.

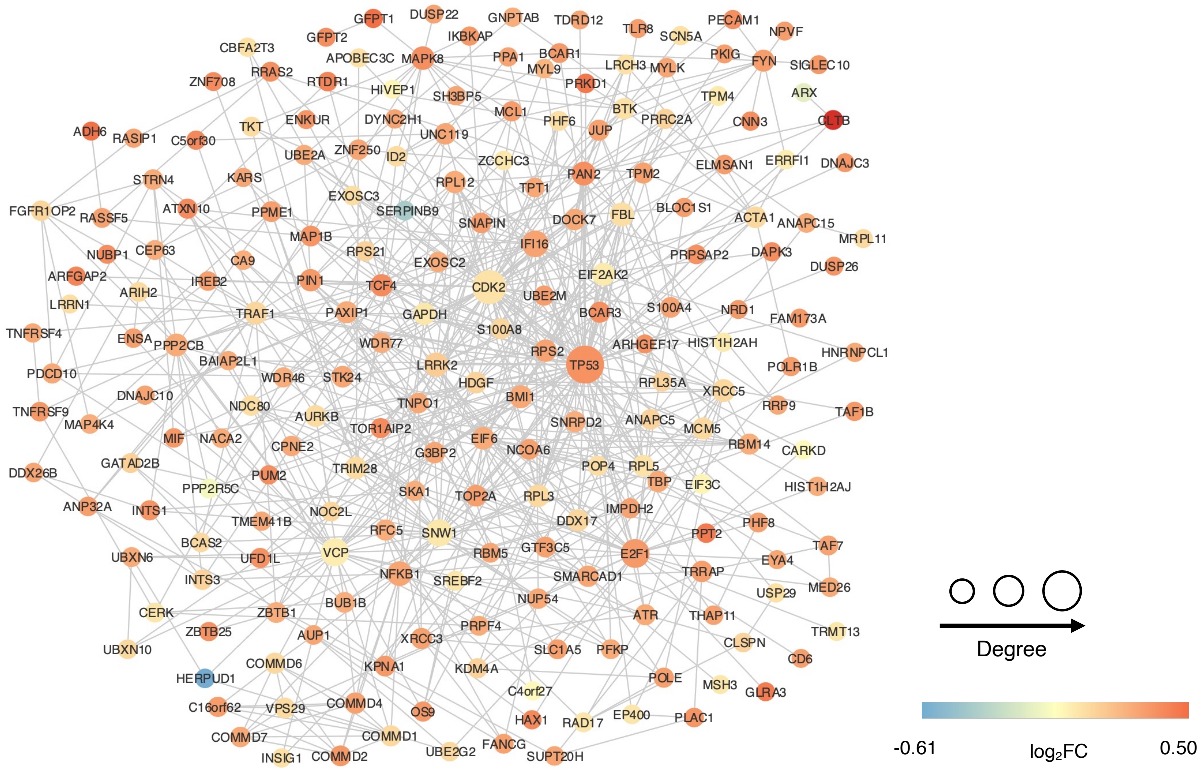

Supplementary Figure 8. Transcriptomic molecular module of the PD-M subtype identified based on gene expression profiles with network-based method, GPSnet. Color indicates log fold change in gene expression, PD-M vs. healthy control. Size of a gene indicates degree (number of connected genes) of the gene in the protein-protein interaction network.

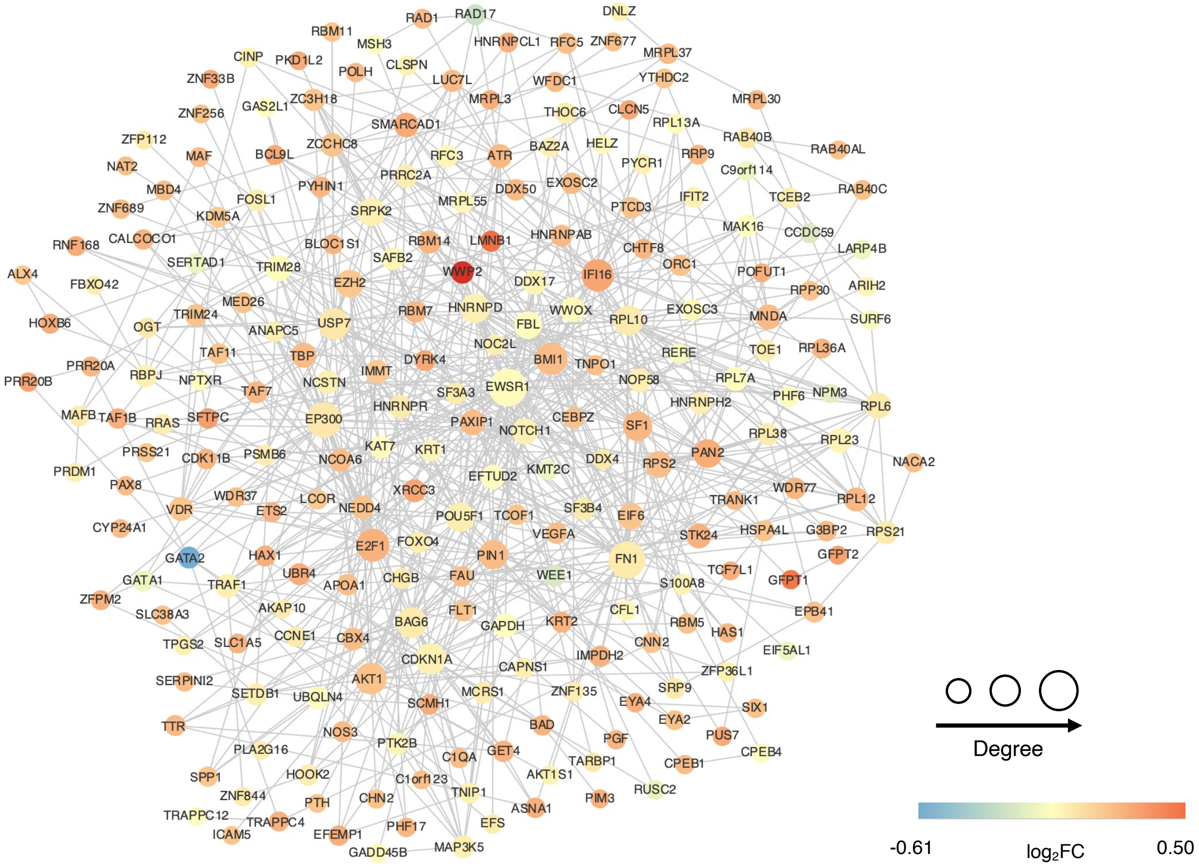

Supplementary Figure 9. Transcriptomic molecular module of the PD-R subtype identified based on gene expression profiles with network-based method, GPSnet. Color indicates log fold change in gene expression, PD-R vs. healthy control. Size of a gene indicates degree (number of connected genes) of the gene in the protein-protein interaction network.

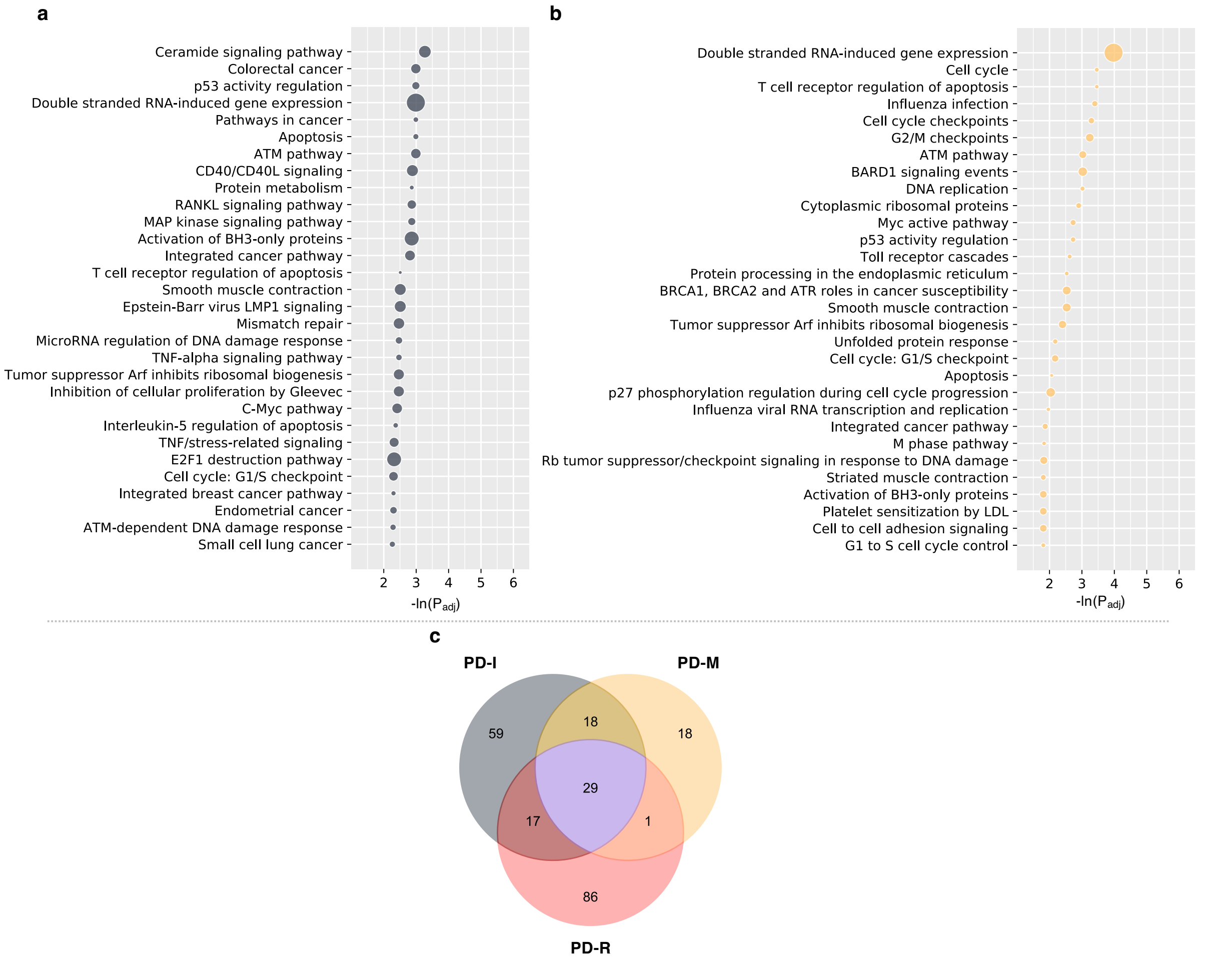

Supplementary Figure 10. Enriched pathways based on transcriptomic molecular module of the subtypes. a. and **b.** Enriched pathways based on transcriptomic molecular module of the PD-I and PD-M subtypes, respectively. **c.** Venn plot showing overlaps of enriched pathways among the three subtypes.

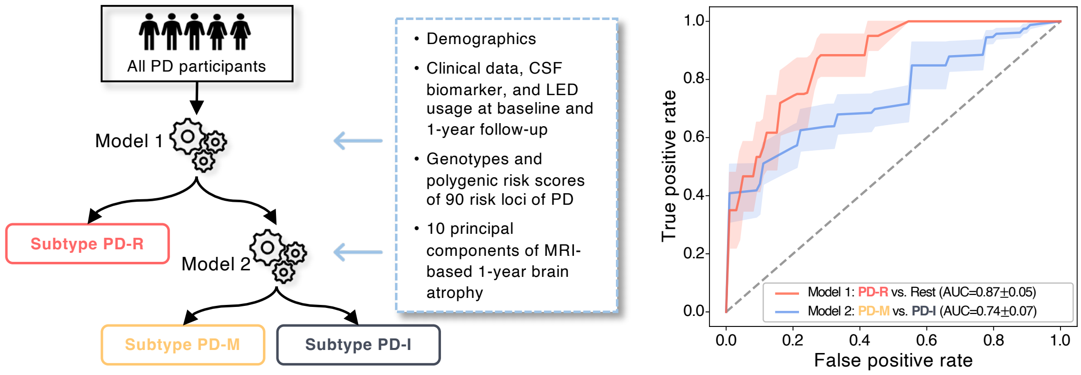

Supplementary Figure 11. Classification model for separating the PD subtypes at early stage. Specifically, we leveraged a cascade framework consisting of two base random forest classifiers: one separating PD-R from the others and another distinguishing PD-I and PD-M. Demographics, genetic profiles, as well as clinical and MRI information within the first year after baseline were used as features to train the model.

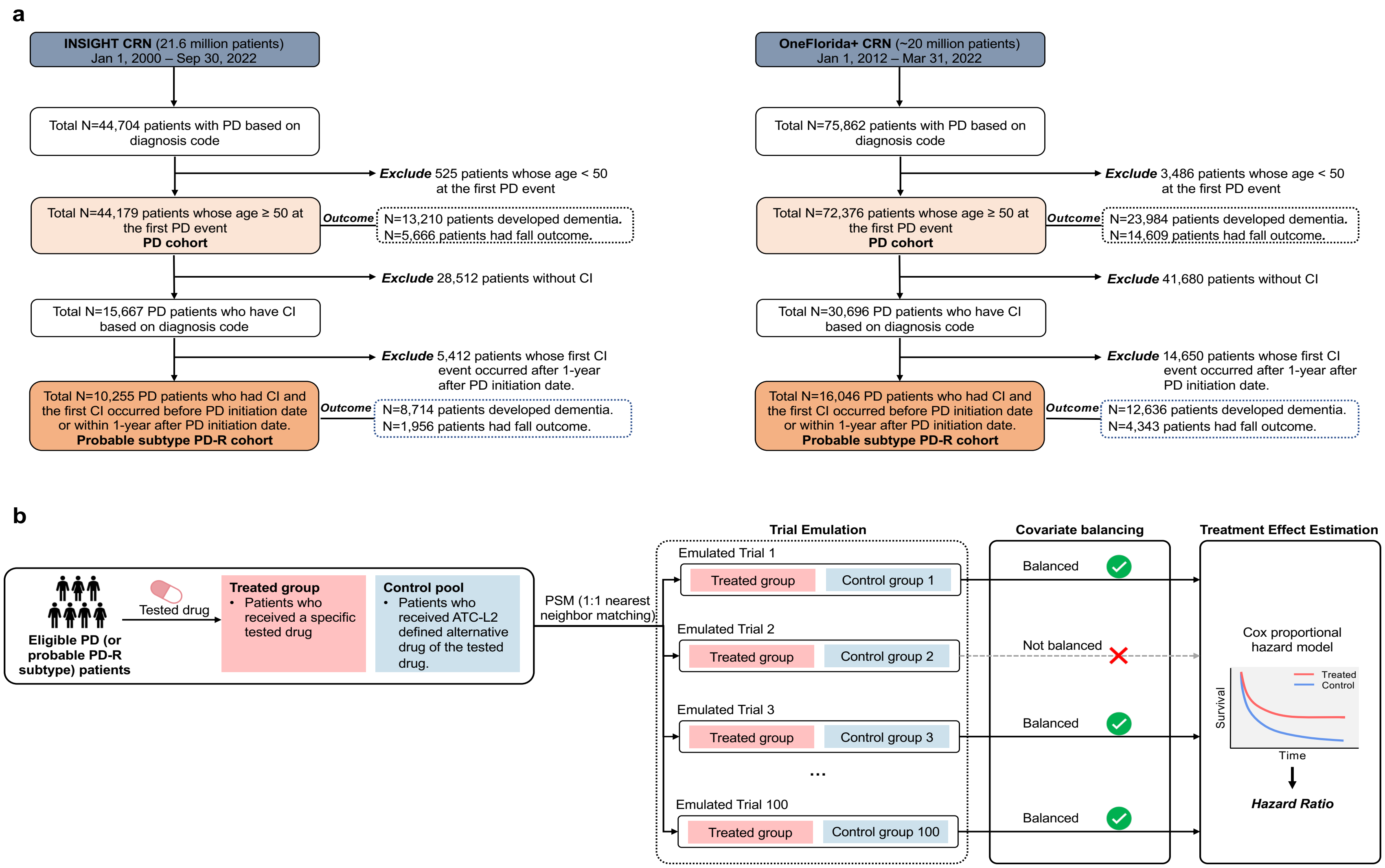

Supplementary Figure 12. Study design of real-world patient data analysis for drug treatment effects estimation. a. Inclusion exclusion criteria. Within each database, we constructed a PD cohort and a probable subtype PD-R cohort as the PD patients who had cognitive impairment (CI) no later than 1 year after 1st PD diagnosis. **b.** Study pipeline of trial emulation based on real-world patient data**.**

Supplementary Tables

Supplementary Table 1. Characteristics and utilization of the studied cohorts

	Development cohort: PPMI			Validation cohort: PDBP		
**Variables**	**HCs**	**SWEDD**	**PDs (*de novo*)**	**HCs**	**Early PDs**	**Other PDs**
**# of participants**	188	61	406	211	210	287
Demographics						
Age at onset, year, mean (SD)	-	-	59.6 (10.0)	-	63.0 (10.0)	57.6 (10.5)
Sex male, N (%)	121 (64.4)	38 (62.3)	266 (65.5)	100 (47.4)	120 (57.1)[a]	182 (63.4)
Race white, N (%)	177 (94.1)	58 (95.1)	384 (94.6)	196 (92.9)	199 (94.8)	261 (90.9)
Symptom duration at baseline, year (SD)	-	-	0.6 (0.7)	-	1.0 (0.8)	8 (6.8)
Family history (%)	6 (3.2)	15 (24.6)	61 (15)	7 (3.3)	28 (13.3)	21 (7.3)[b]
Education history (%)						
< 12 years	5 (2.7)	10 (16.4)	26 (6.4)	2 (0.9)	7 (3.3)	4 (1.4)[c]
12-16 years	110 (58.5)	34 (55.7)	248 (61.1)	141 (66.8)	131 (62.4)	169 (58.9)
≥ 16 years	73 (38.8)	17 (27.9)	132 (32.5)	68 (32.2)	71 (33.8)	113 (39.4)
Utilization						
Training deep progression embedding model	✓	✓	✓	✓	✓	✓
Clustering analysis for subtype identification			✓		✓	

[a]Distribution of sex, PPMI PDs vs. PDBP early PDs, P value < 0.05
[b]Distribution of symptom duration at baseline, PPMI PDs vs. PDBP other PDs, P value < 0.01
[c]Distribution of education history at baseline, PMI PDs vs. PDBP other PDs, P value < 0.05

HC = Healthy controls; PD = Parkinson's disease; PDBP = Parkinson's Disease Biomarkers Program; PPMI = the Parkinson progression marker initiative; SD = standard deviation; SWEDD = subjects with scans without evidence for dopaminergic deficit.

Supplementary Table 2. Clinical variables used for PD subtyping

Category	Data	Description	PPMI	PDBP
Motor assessment	MDS-UPDRS Part II[1]	Self-administered questionnaire of motor experiences of daily living. We used all items.	X	X
	MDS-UPDRS Part III[1]	Motor examination provided by rater. We used all items with medication "OFF".	X	X
	Schwab-England activities of daily living score	Measure of the abilities of individuals living with PD relative to a completely independent situation.	X	X
Non-motor assessment	MDS-UPDRS Part I[1]	Non-motor experiences of daily living. We used all items.	X	X
	Scales for Outcomes in Parkinson's disease-Autonomic (SCOPA-AUT)[2]	The SCOPA-AUT was developed to evaluate autonomic symptoms. We used scores of the 7 domains, including gastrointestinal, urinary, cardiovascular, thermoregulatory, pupillomotor, and sexual.	X	
	Geriatric depression scale (GDS)[3]	Measure of depression in older adults.	X	
	Questionnaire for Impulsive-Compulsive Disorders in Parkinson's disease (QUIP)[4]	Measure of severity of symptoms and support a diagnosis of impulse control disorders and related disorders in PD. We used all items.	X	
	State-Trait Anxiety Inventory (STAI)[5]	The measure of trait and state anxiety. We used the STAI-Strait and STAI-State sub-scores.	X	
	Benton Judgment of Line Orientation (JOLO)[6]	A standardized measure of visuospatial judgment. We used the crude score and MOANS normative scores.	X	
	Hopkins Verbal Learning Test (HVLT)[7]	A memory test with six equivalent forms.	X	
	Letter-number sequencing (LNS)	A subset of Wechsler adult intelligence scale, measuring working memory, attention, mental control	X	
	Montreal Cognitive Assessment (MoCA)[8]	A screening assessment for detecting cognitive impairment. We used the visuospatial, naming, attention, language, delayed recall, abstraction, and verbal fluency sub-scores, and total MoCA score.	X	X
	Semantic verbal-language fluency test[9]	Assessment of semantic knowledge, retrieval ability, and executive functioning. We used the sub-scores in terms of animals, vegetables, and fruits.	X	
	Symbol–Digit Matching (SDM)[10]	A neuropsychological test that examines a person's attention and speed of processing.	X	
	Epworth Sleepiness Score (ESS)[11]	Measure of daytime sleepiness. We used all items.	X	X
	REM sleep behaviour disorder (RBD)[12]	A questionnaire for RBD. We used all items.	X	X
	Cranial Nerve Examination	A kind of neurological examination that is used to identify problems with the cranial nerves. We used the 9 components.	X	
PD medication	Levodopa equivalent daily dose	Levodopa equivalent daily dose	X	

Abbreviations: MDS-UPDRS = Movement Disorders Society–revised Unified Parkinson's Disease Rating Scale.

Supplementary Table 3. 2-year follow-up clinical characteristics by subtypes in the PPMI cohort

**Variables**	**Subtype PD-I (Inching Pace)**	**Subtype PD-M (Moderate Pace)**	**Subtype PD-R (Rapid Pace)**	**P-value[a]**	**Post-hoc[b]**	**P-value adjusted[c]**
**# of participants**	**145**	**207**	**54**	**-**	**-**	**-**
Motor manifestations						
MDS-UPDRS Part II, mean (SD)	6.0 (4.6)	8.2 (4.9)	12.1 (6.0)	<0.001***	All comparisons	<0.001***
MDS-UPDRS Part III, mean (SD)	23.9 (10.6)	27.1 (10.5)	33.4 (13.1)	<0.001***	All comparisons	<0.001**
H&Y Stage, mean (SD)	1.7 (0.6)	1.8 (0.5)	2.1 (0.6)	<0.001**	III vs. rest	0.028*
Schwab and England score, mean (SD)	90.6 (7.2)	89.0 (7.5)	83.5 (9.7)	<0.001***	III vs. rest	<0.001***
Tremor score, mean (SD)	0.5 (0.4)	0.6 (0.4)	0.6 (0.5)	0.006*	I vs. II	0.030*
PIGD score, mean (SD)	0.3 (0.3)	0.3 (0.3)	0.7 (0.6)	<0.001***	III vs. rest	<0.001***
Motor phenotype, N (%)						
Tremor	73 (50.3)	127 (61.4)	18 (33.3)	<0.001***	-	-
Indeterminate	30 (20.7)	48 (23.2)	13 (24.1)			
PIGD	29 (20.0)	32 (15.5)	23 (42.6)			
Non-motor manifestations						
MDS-UPDRS Part I, mean (SD)	6.8 (4.3)	7.4 (5.1)	10.7 (5.4)	<0.001***	III vs. rest	<0.001***
Hallucination, mean (SD)	0.09 (0.32)	0.05 (0.21)	0.24 (0.62)	0.002**	III vs. rest	0.001**
Apathy, mean (SD)	0.3 (0.7)	0.4 (0.7)	0.6 (0.9)	0.088	-	0.205
Pain, mean (SD)	0.8 (0.8)	0.9 (0.9)	1.1 (1.0)	0.076	-	0.016*
Fatigue, mean (SD)	0.6 (0.7)	0.8 (0.9)	1.2 (1.1)	<0.001***	III vs. rest	<0.001**
Sleep, mean (SD)						
Epworth sleepiness score	5.1 (3.4)	7.3 (4.1)	8.5 (4.8)	<0.001***	I vs. rest	<0.001**
REM sleep behavior disorder	3.9 (2.8)	4.7 (3.0)	5.4 (3.4)	0.006*	1 vs. III	0.007*
Sleep phenotype, missing = 1, N (%)						
REM sleep behavior disorder positive	45 (31.0)	92 (44.7)	30 (55.6)	0.002**	-	-
REM sleep behavior disorder negative	100 (69.0)	114 (55.3)	24 (44.4)			
QUIP (Impulse control disorders) , mean (SD)	0.3 (0.7)	0.3 (0.7)	0.3 (0.8)	0.830	-	0.929
Geriatric depression scale, mean (SD)	2.3 (2.8)	2.7 (3.0)	3.4 (2.6)	0.064	-	0.052
Depression phenotype, missing = 1, N (%)						
Normal	122 (84.1)	172 (83.5)	38 (70.4)	0.121	-	-
Mild	12 (8.3)	20 (9.7)	10 (18.5)			
Moderate	6 (4.1)	11 (5.3)	6 (11.1)			
Severe	5 (3.5)	3 (1.5)	0 (0)			
State trait anxiety index, mean (SD)						

State subscore	31.7 (10.4)	32.2 (9.3)	36.3 (11.6)	0.016*	III vs. rest	0.007*
Trait subscore	31.7 (10.3)	32.5 (8.8)	35.3 (10.3)	0.075	-	0.012*
SCOPA autonomic questionnaire, mean (SD)						
Gastrointestinal (up+down)	2.6 (2.3)	2.8 (2.3)	4.3 (2.6)	<0.001***	III vs. rest	<0.001***
Urinary	4.0 (2.5)	4.7 (2.9)	6.5 (7.6)	<0.001**	III vs. rest	0.016*
Cardiovascular	0.6 (0.8)	0.7 (1.1)	0.9 (1.2)	0.083	-	0.128
Thermoregulatory	0.4 (0.8)	0.6 (1.0)	0.4 (0.7)	0.280	-	0.208
Pupillomotor	0.4 (0.7)	0.5 (0.7)	0.6 (0.8)	0.532	-	0.320
Skin	0.8 (1.1)	0.9 (1.0)	0.9 (1.1)	0.727	-	0.312
Sexual	5.0 (6.8)	4.3 (6.0)	5.8 (6.7)	0.274	-	0.559
Total (sum all)	13.9 (8.8)	14.4 (9.2)	19.4 (11.1)	0.001***	III vs. rest	<0.001***
Cognitive function, mean (SD)						
MoCA-visuospatial	4.5 (0.8)	4.3 (0.8)	3.7 (1.5)	<0.001***	III vs. rest	<0.001***
MoCA-naming	2.9 (0.2)	2.9 (0.3)	2.9 (0.4)	0.244	-	0.363
MoCA-attention	5.8 (0.5)	5.6 (0.7)	5.3 (1.0)	<0.001***	III vs. rest	<0.001***
MoCA-language	2.6 (0.5)	2.4 (0.8)	2.0 (1.0)	<0.001***	All comparisons	<0.001***
MoCA-delayed recall	3.7 (1.5)	3.0 (1.7)	2.0 (1.9)	<0.001***	All comparisons	<0.001***
MoCA total score	27.7 (2.4)	26.2 (2.7)	23.6 (4.4)	<0.001***	All comparisons	<0.001***
Benton judgment of line orientation	13.3 (1.8)	12.8 (2.1)	11.4 (3.1)	<0.001***	III vs. rest	<0.001***
HVLT-total recall	25.6 (4.7)	23.5 (5.3)	19.7 (6.1)	<0.001***	All comparisons	<0.001***
HVLT-delayed recall	8.9 (2.5)	8.3 (2.9)	6.1 (3.4)	<0.001***	III vs. rest	<0.001***
HVLT-discrimination recognition	11.0 (2.3)	10.7 (2.2)	9.7 (3.1)	0.005**	III vs. rest	0.047
HVLT-retention	0.9 (0.2)	0.9 (0.2)	0.7 (0.3)	<0.001***	III vs. rest	0.010*
LNS	11.2 (2.6)	10.2 (2.5)	8.4 (3.4)	<0.001***	All comparisons	<0.001***
Semantic fluency	53.7 (13.4)	48.3 (11.9)	39.4 (11.2)	<0.001***	All comparisons	<0.001***
Symbol digit test	48 (9.6)	43.9 (9.7)	38.0 (10.8)	<0.001***	All comparisons	<0.001***
Cognitive phenotype, missing = 7, N (%)						
Normal	133 (96.4)	193 (93.2)	39 (72.2)			
MCI	4 (2.8)	9 (4.4)	9 (16.7)	<0.001***	-	-
Dementia	1 (0.7)	5 (2.4)	6 (11.1)			

[a] P-values were calculated using ANOVA (for continuous variables) and χ^2 test (for categorical variables) where appropriate.
[b] Post-hoc analysis was performed using the Tukey HSD test when the ANOVA P-value < 0.05.
[c] ANCOVA was used to calculate p-values (for continuous variables) adjusting for age and sex.
Multiple correction was conducted by controlling false discovery rate (FDR). * FDR adjusted P-value< 0.05; ** FDR adjusted P-value< 0.01; *** FDR adjusted P-value< 0.001.

HVLT = Hopkins Verbal Learning Test; MCI = mild cognitive impairment; MDS-UPDRS = Movement Disorders Society–revised Unified Parkinson's Disease Rating Scale; MoCA = Montreal Cognitive Assessment; PIGD = postural instability and gait disorder; PPMI = the Parkinson's Progression Markers Initiative; SCOPA = Scales for Outcomes in Parkinson's Disease.

Supplementary Table 4. 5-year follow-up clinical characteristics by subtypes in the PPMI cohort

Variables	Subtype PD-I (Inching Pace)	Subtype PD-M (Moderate Pace)	Subtype PD-R (Rapid Pace)	P-value[a]	Post-hoc[b]	P-value adjusted[c]
**# of participants**	**145**	**207**	**54**	**-**	**-**	**-**
Motor manifestations						
MDS-UPDRS Part II, mean (SD)	5.7 (4.3)	11.1 (5.6)	16.5 (10.0)	<0.001***	All comparisons	<0.001***
MDS-UPDRS Part III, mean (SD)	23.0 (9.5)	32.5 (12.9)	38.9 (15.8)	<0.001***	All comparisons	<0.001***
H&Y Stage, mean (SD)	1.9 (0.4)	2.0 (0.4)	2.5 (0.9)	<0.001***	III vs. rest	<0.001***
Schwab and England score, mean (SD)	90.5 (6.6)	84.1 (9.3)	70.9 (23.6)	<0.001***	All comparisons	<0.001***
Tremor score, mean (SD)	0.5 (0.4)	0.7 (0.5)	0.6 (0.5)	<0.001**	I vs. II	0.003**
PIGD score, mean (SD)	0.3 (0.3)	0.5 (0.4)	1.2 (1.0)	<0.001***	All comparisons	<0.001***
Motor phenotype, N (%)						
Tremor	54 (37.2)	102 (49.3)	8 (14.8)	0.004**	-	-
Indeterminate	24 (16.6)	49 (23. 7)	6 (11.1)			
PIGD	21(14.5)	56 (27.1)	20 (37.0)			
Non-motor manifestations						
MDS-UPDRS Part I, mean (SD)	6.5 (4.7)	9.8 (5.6)	14.3 (9.0)	<0.001***	All comparisons	<0.001***
Hallucination, mean (SD)	0.1 (0.3)	0.2 (0.4)	0.4 (1.1)	0.002**	III vs. rest	0.005**
Apathy, mean (SD)	0.2 (0.5)	0.5 (0.8)	0.9 (1.2)	<0.001***	All comparisons	<0.001***
Pain, mean (SD)	0.6 (0.9)	1.0 (1.1)	1.4 (1.1)	0.002**	I vs. rest	<0.001***
Fatigue, mean (SD)	0.7 (0.8)	1.1 (1.0)	1.6 (1.2)	<0.001***	All comparisons	<0.001***
Sleep, mean (SD)						
Epworth sleepiness score	5.4 (3.9)	8.5 (4.4)	10.2 (5.7)	<0.001***	I vs. rest	<0.001***
REM sleep behavior disorder	3.7 (2.9)	5.3 (3.1)	5.7 (3.6)	<0.001***	I vs. rest	<0.001**
Sleep phenotype, missing = 1, N (%)						
REM sleep behavior disorder positive	51 (35.2)	117 (56.8)	30 (55.6)	<0.001***	-	-
REM sleep behavior disorder negative	94 (64.8)	89 (43.2)	24 (44.4)			
QUIP (Impulse control disorders) , mean (SD)	0.3 (0.7)	0.5 (0.9)	0.2 (0.4)	0.028*		0.054
Geriatric depression scale, mean (SD)	1.6 (1.9)	3.0 (2.6)	4.9 (4.2)	<0.001***	All comparisons	<0.001***
Depression phenotype, missing = 1, N (%)						
Normal	126 (86.9)	160 (77.7)	30 (55.6)	<0.001***	-	-
Mild	13 (9.0)	30 (14.6)	10 (18.5)			
Moderate	2 (1.4)	15 (7.3)	11 (20.4)			
Severe	4 (2.7)	1 (0.5)	3 (5.6)			

State trait anxiety index, mean (SD)						
State subscore	28.8 (8.9)	33.0 (9.5)	36.1 (13.4)	<0.001***	I vs. rest	<0.001***
Trait subscore	29.5 (9.2)	33.7 (9.7)	36.8 (13.5)	<0.001***	I vs. rest	<0.001***
SCOPA autonomic questionnaire, mean (SD)						
Gastrointestinal (up+down)	2.7 (2.2)	3.8 (2.5)	5.1 (3.7)	<0.001***	All comparisons	<0.001***
Urinary	4.4 (2.7)	5.8 (5.3)	6.7 (4.8)	0.017*	I vs. rest	0.111
Cardiovascular	0.5 (0.9)	0.8 (1.2)	1.4 (1.8)	0.001**	III vs. rest	0.002**
Thermoregulatory	0.5 (1.1)	0.8 (1.2)	0.4 (0.7)	0.054	-	0.032*
Pupillomotor	0.4 (0.7)	0.6 (0.8)	0.8 (1.0)	0.048	-	0.062
Skin	0.7 (1.0)	1.2 (1.2)	1.5 (1.9)	<0.001***	I vs. rest	<0.001***
Sexual	4.5 (6.3)	5.2 (6.4)	6.7 (7.4)	0.248	-	0.085
Total (sum all)	13.7 (8.7)	18.3 (11.1)	22.5 (15.2)	<0.001***	I vs. rest	<0.001***
Cognitive function, mean (SD)						
MoCA-visuospatial	4.6 (0.7)	4.2 (1.1)	3.6 (1.6)	<0.001***	All comparisons	<0.001***
MoCA-naming	3 (0.2)	2.9 (0.2)	2.8 (0.6)	0.041	III vs. rest	0.054
MoCA-attention	5.7 (0.5)	5.5 (0.8)	4.8 (1.4)	<0.001***	III vs. rest	<0.001***
MoCA-language	2.6 (0.6)	2.5 (0.7)	2.2 (0.9)	0.013*	I vs. III	0.028*
MoCA-delayed recall	4.3 (1.1)	3.3 (1.6)	1.9 (1.8)	<0.001***	All comparisons	<0.001***
MoCA total score	28.3 (1.7)	26.5 (3.2)	23.0 (5.5)	<0.001***	All comparisons	<0.001***
Benton judgment of line orientation	13.0 (2)	12.3 (2.2)	11.0 (2.8)	<0.001***	All comparisons	<0.001***
HVLT-total recall	27.6 (5.1)	24.1 (6.3)	17.2 (4.9)	<0.001***	All comparisons	<0.001***
HVLT-delayed recall	10 (2.4)	8.4 (3.1)	4.9 (3.1)	<0.001***	All comparisons	<0.001***
HVLT-discrimination recognition	11.3 (1.1)	10.5 (2.0)	8.9 (2.5)	<0.001***	All comparisons	<0.001***
HVLT-retention	0.9 (0.2)	0.9 (0.2)	0.7 (0.4)	<0.001***	All comparisons	<0.001***
LNS	11.5 (2.6)	9.7 (2.8)	8.0 (3.4)	<0.001***	All comparisons	<0.001***
Semantic fluency	55.6 (12.4)	47.1 (11.5)	35.3 (13.4)	<0.001***	All comparisons	<0.001***
Symbol digit test	50.4 (10.7)	44.1 (10.7)	39.4 (15.3)	<0.001***	I vs. rest	<0.001***
Cognitive phenotype, missing = 7, N (%)						
Normal	134 (97.1)	185 (89.4)	31 (51.4)	<0.001***	-	-
MCI	3 (2.2)	16 (7.7)	8 (14.8)			
Dementia	1 (0.7)	6 (2.9)	15 (27.8)			

[a] P-values were calculated using ANOVA (for continuous variables) and χ^2 test (for categorical variables) where appropriate.
[b] Post-hoc analysis was performed using the Tukey HSD test when the ANOVA P-value < 0.05.
[c] ANCOVA was used to calculate p-values (for continuous variables) adjusting for age, sex, and levodopa equivalent daily dose.
Multiple correction was conducted by controlling false discovery rate (FDR). * FDR adjusted P-value< 0.05; ** FDR adjusted P-value< 0.01; *** FDR adjusted P-value<

0.001.

HVLT = Hopkins Verbal Learning Test; MCI = mild cognitive impairment; MDS-UPDRS = Movement Disorders Society–revised Unified Parkinson's Disease Rating Scale; MoCA = Montreal Cognitive Assessment; PIGD = postural instability and gait disorder; PPMI = the Parkinson's Progression Markers Initiative; SCOPA = Scales for Outcomes in Parkinson's Disease.

Supplementary Table 5. Demographics and baseline clinical characteristics by subtypes in the PDBP cohort

Variables	Subtype PD-I (Inching Pace)	Subtype PD-M (Moderate Pace)	Subtype PD-R (Rapid Pace)	P-value[a]	Post-hoc[b]	P-value adjusted[c]
**# of participants**	**55**	**72**	**49**	**-**	**-**	**-**
Age at onset, year, mean (SD)	58.6 (9.8)	64.8 (9.3)	65.2 (9.8)	<0.001	I vs. rest	-
Sex male, N (%)	28 (50.9)	43 (59.7)	32 (65.3)	0.335	-	-
Race white, N (%)	54 (98.2)	66 (91.7)	47 (95.9)	0.232	-	-
Symptom duration, year, mean (SD)	0.9 (0.8)	0.8 (0.7)	1.1 (0.8)	0.183	-	-
Family history, N (%)	7 (12.7)	10 (13.4)	8 (16.3)	0.896	-	-
Education history, N (%)						
Less than 12 years	-	2 (2.8)	1 (2.0)	0.257	-	-
12-16 years	32 (58.2)	50 (69.4)	27 (55.1)			
Greater than 16 years	23 (41.8)	20 (27.8)	20 (40.8)			
Motor manifestations						
MDS-UPDRS Part II, mean (SD)	3.9 (2.9)	5.6 (4.7)	9.5 (6.0)	<0.001***	III vs. rest	<0.001***
MDS-UPDRS Part III, mean (SD)	14.9 (8.9)	18.1 (8.9)	24.4 (10.4)	<0.001***	III vs. rest	0.012*
H&Y Stage, mean (SD)	1.7 (0.5)	1.8 (0.5)	2.1 (0.5)	<0.001***	III vs. rest	<0.001***
Schwab and England score, mean (SD)	94.4 (5.0)	91.9 (7.8)	88.2 (6.7)	<0.001***	III vs. rest	<0.001***
Tremor score, mean (SD)	0.4 (0.4)	0.5 (0.4)	0.4 (0.3)	0.169	-	0.186
PIGD score, mean (SD)	0.2 (0.2)	0.3 (0.2)	0.6 (0.5)	<0.001***	III vs. rest	<0.001***
Motor phenotype, N (%)						
Tremor	37 (67.3)	48 (66.7)	18 (36.7)	0.002**	III vs. rest	-
Indeterminate	4 (7.2)	8 (11.1)	4 (8.2)			
PIGD	14 (25.5)	16 (22.2)	27 (55.1)			
Non-motor manifestations						
MDS-UPDRS Part I, mean (SD)	4.7 (3.3)	6.2 (4.4)	9.1 (5.0)	<0.001***	III vs. rest	<0.001***
Hallucination, mean (SD)	0.02 (0.12)	0.03 (0.17)	0.14 (0.35)	0.009*	III vs. rest	0.010*
Apathy, mean (SD)	0.2 (0.4)	0.2 (0.5)	0.4 (0.8)	0.201	-	0.170
Pain, mean (SD)	0. 8 (0.9)	0.7 (0.7)	1.0 (1.1)	0.116	-	0.076
Fatigue, mean (SD)	0.7 (0.6)	0.8 (0.8)	1.1 (0.9)	0.039	I vs. III	0.037
Sleep, mean (SD)						
Epworth sleepiness score	5.1 (3.2)	5.4 (3.2)	8.3 (5.1)	<0.001***	I vs. III	<0.001***
REM sleep behavior disorder	0.1 (0.8)	0.08 (0.4)	0.1 (0.7)	0.885	-	0.817
Cognitive function, mean (SD)						
MoCA-language	2.5 (0.8)	2.4 (0.7)	2.3 (0.8)	0.401	-	0.575

MoCA total score	27.1 (2.2)	26.2 (2.4)	26.2 (2.5)	0.050	-	0.361

[a] P-values were calculated using ANOVA (for continuous variables) and χ^2 test (for categorical variables) where appropriate.
[b] Post-hoc analysis was performed using the Tukey HSD test when the ANOVA P-value < 0.05.
[c] ANCOVA was used to calculate p-values (for continuous variables) adjusting for age and sex.
Multiple correction was conducted by controlling false discovery rate (FDR). * FDR adjusted P-value< 0.05; ** FDR adjusted P-value< 0.01; *** FDR adjusted P-value< 0.001.

MDS-UPDRS = Movement Disorders Society–revised Unified Parkinson's Disease Rating Scale; MoCA = Montreal Cognitive Assessment; PDBP = the Parkinson Disease Biomarkers Program; PIGD = postural instability and gait disorder.

Supplementary Table 6. 2-year follow-up clinical characteristics by subtypes in the PDBP cohort

**Variables**	**Subtype PD-I (Inching Pace)**	**Subtype PD-M (Moderate Pace)**	**Subtype PD-R (Rapid Pace)**	**P-value[a]**	**Post-hoc[b]**	**P-value adjusted[c]**
**# of participants**	**55**	**72**	**49**	**-**	**-**	**-**
Motor manifestations						
MDS-UPDRS Part II, mean (SD)	4.2 (3.6)	6.5 (4.4)	14.6 (8.5)	<0.001***	III vs. rest	<0.001***
MDS-UPDRS Part III, mean (SD)	13.5 (5.8)	17.1 (7.8)	27.3 (12.4)	<0.001***	III vs. rest	<0.001***
H&Y Stage, mean (SD)	1.9 (0.4)	1.9 (0.3)	2.0 (0.7)	0.572	-	0.708
Schwab and England score, mean (SD)	93.3 (6)	92.2 (11.7)	81.2 (17.7)	<0.001***	III vs. rest	<0.001***
Tremor score, mean (SD)	0.4 (0.3)	0.5 (0.3)	0.5 (0.3)	0.095	-	0.123
PIGD score, mean (SD)	0.1 (0.2)	0.2 (0.2)	0.6 (0.7)	<0.001***	III vs. rest	<0.001***
Motor phenotype, N (%)						
Tremor	27 (49.1)	52 (72.2)	21 (42.9)	0.005**	-	-
Indeterminate	10 (18.2)	2 (2.8)	2 (4.1)			
PIGD	8 (14.6)	6 (8.3)	19 (38.8)			
Non-motor manifestations						
MDS-UPDRS Part I, mean (SD)	4.9 (3.8)	7.7 (5.3)	11.4 (6.3)	<0.001***	All comparisons	<0.001***
Hallucination, mean (SD)	0.04 (0.21)	0.03 (0.18)	0.24 (0.73)	0.036*	II vs. III	0.022*
Apathy, mean (SD)	0.1 (0.3)	0.1 (0.4)	0.4 (0.7)	0.002**	III vs. rest	0.002**
Pain, mean (SD)	0.6 (0.7)	0.8 (0.9)	1.3 (1.2)	0.005**	I vs. III	0.002**
Fatigue, mean (SD)	0.6 (0.8)	1.1 (1.1)	1.2 (1.0)	0.015*	I vs. II	0.004**
Sleep, mean (SD)						
Epworth sleepiness score	5.2(3.4)	5.6 (4.4)	10.1 (4.3)	<0.001***	III vs. rest	<0.001***
REM sleep behavior disorder	0.1 (0.9)	0.05 (0.3)	0.2 (0.8)	0.656	-	0.642
Cognitive function, mean (SD)						
MoCA-language	2.5 (0.7)	2.4 (0.8)	2.1 (0.9)	0.053	-	0.097
MoCA total score	27.6 (2.1)	26.1 (2.8)	24.5 (5.4)	<0.001***	I vs. III	0.014*

[a] P-values were calculated using ANOVA (for continuous variables) and χ^2 test (for categorical variables) where appropriate.
[b] Post-hoc analysis was performed using the Tukey HSD test when the ANOVA P-value < 0.05.
[c] ANCOVA was used to calculate p-values (for continuous variables) adjusting for age and sex.
Multiple correction was conducted by controlling false discovery rate (FDR). * FDR adjusted P-value< 0.05; ** FDR adjusted P-value< 0.01; *** FDR adjusted P-value< 0.001.

MDS-UPDRS = Movement Disorders Society–revised Unified Parkinson's Disease Rating Scale; MoCA = Montreal Cognitive Assessment; PIGD = postural instability and

gait disorder.

Supplementary Table 7. 4-year follow-up clinical characteristics by subtypes in the PDBP cohort

Variables	Subtype PD-I (Inching Pace)	Subtype PD-M (Moderate Pace)	Subtype PD-R (Rapid Pace)	P-value[a]	Post-hoc[b]	P-value adjusted[c]
**# of participants**	**55**	**72**	**49**	**-**	**-**	**-**
Motor manifestations						
MDS-UPDRS Part II, mean (SD)	2.7 (1.7)	8 (4.0)	16.2 (6.6)	<0.001***	All comparisons	<0.001***
MDS-UPDRS Part III, mean (SD)	17.9 (7.8)	21.8 (5.8)	31.7 (9.9)	0.002**	III vs. rest	0.003**
H&Y Stage, mean (SD)	2.0 (0.0)	2.0 (0.0)	2.4 (0.7)	0.022*	III vs. rest	0.019*
Schwab and England score, mean (SD)	95 (5.3)	90.8 (4.9)	76.7 (18.7)	0.002**	III vs. rest	0.003**
Tremor score, mean (SD)	0.4 (0.3)	0.4 (0.4)	0.5 (0.2)	0.714	-	0.598
PIGD score, mean (SD)	0.1 (0.1)	0.4 (0.3)	1.1 (0.5)	<0.001***	III vs. rest	<0.001***
Motor phenotype, N (%)						
Tremor	8 (14.6)	5 (7.0)	2 (4.1)	0.056	-	-
Indeterminate	1 (1.9)	4 (5.6)	1 (2.0)			
PIGD	1 (1.9)	4 (5.6)	6 (12.2)			
Non-motor manifestations						
MDS-UPDRS Part I, mean (SD)	4.2 (3.0)	8.4 (2.9)	13.6 (7.3)	<0.001***	III vs. rest	<0.001***
Hallucination, mean (SD)	-	-	0.6 (0.7)	-	-	-
Apathy, mean (SD)	0.2 (0.4)	0.1 (0.3)	0.3 (0.5)	0.337	-	0.288
Pain, mean (SD)	0.5 (0.5)	1.4 (1.3)	1.6 (1.1)	0.070	-	0.09
Fatigue, mean (SD)	0.6 (0.7)	1.1 (1.0)	1.2 (1.0)	0.282	-	0.292
Sleep, mean (SD)						
Epworth sleepiness score	4.4 (2.2)	5.8 (3.1)	9.9 (3.5)	0.001**	III vs. rest	<0.001***
REM sleep behavior disorder	-	-	-	-	-	-
Cognitive function, mean (SD)						
MoCA-language	2.8 (0.4)	2.5 (0.9)	2.1 (1.1)	0.209	-	0.309
MoCA total score	28.2 (1.9)	24.4 (4.4)	24.9 (3.6)	0.039	I vs. II	0.096

[a] P-values were calculated using ANOVA (for continuous variables) and χ^2 test (for categorical variables) where appropriate.
[b] Post-hoc analysis was performed using the Tukey HSD test when the ANOVA P-value < 0.05.
[c] ANCOVA was used to calculate p-values (for continuous variables) adjusting for age and sex.
Multiple correction was conducted by controlling false discovery rate (FDR). * FDR adjusted P-value< 0.05; ** FDR adjusted P-value< 0.01; *** FDR adjusted P-value< 0.001.

MDS-UPDRS = Movement Disorders Society–revised Unified Parkinson's Disease Rating Scale; MoCA = Montreal Cognitive Assessment; PIGD = postural instability and

gait disorder.

Supplementary Table 8. Annual progression rates in clinical manifestations and CSF biomarkers by subtypes assessed by linear mixed effects models in the PDB cohort

Variable	Subtype PD-I (Inching Pace)		Subtype PD-M (Moderate Pace)		Subtype PD-R (Rapid Pace)	
	β	**P value**	***β***	**P value**	***β***	**P value**
Motor manifestations						
MDS-UPDRS Part II	0.10 (-0.20, 0.40)	0.530	1.01 (0.67, 1.36)	<0.001***	2.53 (1.40, 3.67)	<0.001***
MDS-UPDRS Part III	-0.30 (-1.07, 0.47)	0.444	1.12 (0.46, 1.78)	0.002**	2.70 (1.70, 3.70)	<0.001***
H&Y Stage	0.04 (-0.00, 0.08)	0.078	0.05 (0.02, 0.08)	0.007*	0.10 (0.01, 0.19)	0.042
Schwab and England score	-0.48 (-1.10, 0.11)	0.117	-0.28 (-1.10, 0.53)	0.505	-4.05 (-5.92, -2.18)	<0.001***
Tremor score	-0.01 (-0.03, 0.02)	0.706	0.01 (-0.02, 0.03)	0.465	0.03 (-0.00, 0.06)	0.095
PIGD score	-0.03 (-0.04, -0.01)	<0.001*	0.02 (-0.00, 0.041)	0.110	0.14 (0.06, 0.22)	0.001**
Non-motor manifestations						
MDS-UPDRS Part I	0.11 (-0.10, 0.32)	0.333	0.85 (0.53, 1.16)	<0.001***	1.36 (0.91, 1.82)	<0.001***
Hallucination	0.01 (-0.01, 0.03)	0.422	0.01 (-0.01, 0.02)	0.377	0.06 (0.01, 0.12)	0.031*
Apathy	-0.00 (-0.05, 0.04)	0.853	0.04 (-0.01, 0.09)	0.162	0.05 (-0.02, 0.13)	0.187
Pain	-0.03 (-0.09, 0.04)	0.453	0.13 (0.07, 0.20)	<0.001***	0.14 (0.03, 0.24)	0.013*
Fatigue	-0.02 (-0.08, 0.03)	0.440	0.09 (0.02, 0.16)	0.023	0.13 (0.06, 0.20)	0.002**
Sleep						
Epworth sleepiness score	0.12 (-0.11, 0.34)	0.327	0.17 (-0.14, 0.47)	0.294	0.69 (0.23, 1.10)	0.005**
REM sleep behavior disorder[a]	-	-	-	-	-	-
Cognitive function						
MoCA-language	0.03 (-0.05, 0.11)	0.469	0.01 (-0.07, 0.08)	0.845	-0.16 (-0.26, -0.06)	0.005
MoCA total score	0.02 (-0.15, 0.18)	0.836	-0.15 (-0.4, 0.09)	0.214	-1.10 (-1.62, -0.57)	<0.001***

[a] The values in REM sleep behavior disorders are so sparse that the corresponding beta is not available.
Multiple correction was conducted by controlling false discovery rate (FDR). * FDR adjusted P-value< 0.05; ** FDR adjusted P-value< 0.01; *** FDR adjusted P-value< 0.001.

MDS-UPDRS = Movement Disorders Society–revised Unified Parkinson's Disease Rating Scale; MoCA = Montreal Cognitive Assessment; PDBP = the Parkinson Disease Biomarkers Program; PIGD = postural instability and gait disorder.

Supplementary Table 9. Baseline CSF biomarkers by subtypes in the PPMI cohort

**Biomarker**	**HC**	**PD-I, mean (SD)**	**PD-M, mean (SD)**	**PD-R, mean (SD)**	**P values[a]**					
					HC vs. PD-I	HC vs. PD-M	HC vs. PD-R	PD-M vs. PD-I	PD-R vs. PD-I	PD-R vs. PD-M
α-synuclein	1704.491 (752.640)	1607.109 (734.891)	1487.264 (659.634)	1357.906 (505.550)	0.020	0.001	0.218	0.153	0.011	0.068
Aβ-42	1025.042 (498.628)	970.128 (463.830)	905.070 (386.737)	781.500 (351.559)	0.148	0.398	0.912	0.227	0.009	0.045
P-tau	16.845 (8.412)	14.342 (5.403)	14.014 (5.107)	14.959 (6.352)	0.013	<0.001	0.023	0.571	0.767	0.760
T-tau	190.283 (79.901)	169.613 (59.369)	163.533 (53.990)	170.126 (66.713)	0.014	<0.001	0.017	0.319	0.437	0.933
Aβ-42/T-tau	5.578 (1.649)	5.713 (1.480)	5.635 (1.572)	4.906 (1.885)	0.479	<0.001	0.009	0.962	0.022	0.051
Aβ-42/α-synuclein	0.636 (0.221)	0.637 (0.219)	0.659 (0.282)	0.593 (0.208)	0.182	<0.001	0.218	0.311	0.340	0.402
P-tau/α-synuclein	0.010 (0.002)	0.009 (0.002)	0.010 (0.002)	0.011 (0.003)	0.821	0.742	0.004	0.072	0.001	0.007
P-tau/T-tau	0.087 (0.007)	0.084 (0.008)	0.085 (0.007)	0.087 (0.008)	0.057	0.228	0.140	0.215	0.114	0.145
T-tau/α-synuclein	0.116 (0.026)	0.113 (0.028)	0.117 (0.029)	0.128 (0.026)	0.664	0.400	0.017	0.219	0.015	0.036
Aβ-42/P-tau	64.795 (20.617)	68.010 (17.636)	66.679 (20.169)	56.961 (22.692)	0.207	<0.001	0.006	0.841	0.010	0.037

[a]ANCOVA was used to calculate p-values adjusting for age and sex.

Aβ-42 = the 42 amino acid form of amyloid-β; CSF = cerebrospinal fluid.

Supplementary References

1 Goetz, C. G. *et al.* Movement Disorder Society-sponsored revision of the Unified Parkinson's Disease Rating Scale (MDS-UPDRS): Scale presentation and clinimetric testing results. *Movement Disorders* **23**, 2129-2170 (2008).

2 Visser, M., Marinus, J., Stiggelbout, A. M. & Van Hilten, J. J. Assessment of autonomic dysfunction in Parkinson's disease: The SCOPA-AUT. *Movement Disorders* **19**, 1306-1312 (2004).

3 Sheikh, J. I. & Yesavage, J. A. Geriatric Depression Scale (GDS): recent evidence and development of a shorter version. *Clinical Gerontologist: The Journal of Aging and Mental Health* (1986).

4 Weintraub, D. *et al.* Validation of the questionnaire for impulsive-compulsive disorders in Parkinson's disease. *Movement Disorders* **24**, 1461-1467 (2009).

5 Spielberger, C. D. State-Trait Anxiety Inventory. In *The Corsini Encyclopedia of Psychology* 1-1.

6 Benton, A. L., Varney, N. R. & Hamsher, K. d. Visuospatial Judgment: A Clinical Test. *Archives of Neurology* **35**, 364-367 (1978).

7 Shapiro, A. M., Benedict, R. H. B., Schretlen, D. & Brandt, J. Construct and Concurrent Validity of the Hopkins Verbal Learning Test – Revised. *The Clinical Neuropsychologist* **13**, 348-358 (1999).

8 Nasreddine, Z. S. *et al.* The Montreal Cognitive Assessment, MoCA: A Brief Screening Tool For Mild Cognitive Impairment. *Journal of the American Geriatrics Society* **53**, 695-699 (2005).

9 Gladsjo, J. A. *et al.* Norms for Letter and Category Fluency: Demographic Corrections for Age, Education, and Ethnicity. *Assessment* **6**, 147-178 (1999).

10 Smith, A. *Symbol digit modalities test.* (Western Psychological Services Los Angeles, 1973).

11 Johns, M. W. A New Method for Measuring Daytime Sleepiness: The Epworth Sleepiness Scale. *Sleep* **14**, 540-545 (1991).

12 Stiasny-Kolster, K. *et al.* The REM sleep behavior disorder screening questionnaire—A new diagnostic instrument. *Movement Disorders* **22**, 2386-2393 (2007).
