# Supplementary material for: Identification of Parkinson’s disease PACE subtypes and repurposing treatments through integrative analyses of multimodal data
Source: NPJ Digit Med. 2024 Jul 9;7:184. doi: 10.1038/s41746-024-01175-9 (PMC11233682; doi:10.1038/s41746-024-01175-9)
Supplement: Supplementary file 2 — Reporting Summary [file 41746_2024_1175_MOESM2_ESM.pdf]

nature portfolio

Corresponding author(s): Fei Wang

Last updated by author(s): Dec 23, 2023

Reporting Summary

Nature Portfolio wishes to improve the reproducibility of the work that we publish. This form provides structure for consistency and transparency in reporting. For further information on Nature Portfolio policies, see our Editorial Policies and the Editorial Policy Checklist.

Statistics

For all statistical analyses, confirm that the following items are present in the figure legend, table legend, main text, or Methods section.

n/a	Confirmed	
☐	☒	The exact sample size (*n*) for each experimental group/condition, given as a discrete number and unit of measurement
☐	☒	A statement on whether measurements were taken from distinct samples or whether the same sample was measured repeatedly
☐	☒	The statistical test(s) used AND whether they are one- or two-sided<br>*Only common tests should be described solely by name; describe more complex techniques in the Methods section.*
☐	☒	A description of all covariates tested
☐	☒	A description of any assumptions or corrections, such as tests of normality and adjustment for multiple comparisons
☐	☒	A full description of the statistical parameters including central tendency (e.g. means) or other basic estimates (e.g. regression coefficient) AND variation (e.g. standard deviation) or associated estimates of uncertainty (e.g. confidence intervals)
☐	☒	For null hypothesis testing, the test statistic (e.g. *F*, *t*, *r*) with confidence intervals, effect sizes, degrees of freedom and *P* value noted<br>*Give P values as exact values whenever suitable.*
☒	☐	For Bayesian analysis, information on the choice of priors and Markov chain Monte Carlo settings
☒	☐	For hierarchical and complex designs, identification of the appropriate level for tests and full reporting of outcomes
☒	☐	Estimates of effect sizes (e.g. Cohen's *d*, Pearson's *r*), indicating how they were calculated

Our web collection on statistics for biologists contains articles on many of the points above.

Software and code

Policy information about availability of computer code

Data collection	Data from the PPMI can be download from the official web site (http://www.ppmi-info.org); Data from the PDBP can be download from AMP-PD platform (http://amp-pd.org). INSIGHT and OneFlorida+ databases collected patient data following OMOP data standard. SAS Enterprise Guide Version 7.1 was used to collect the NSIGHT and OneFlorida+ data. Information of the INSIGHT database is available at https://insightcrn.org. Request of INSIGHT data can be sent via: https://nyc-cdrn.atlassian.net/servicedesk/customer/portal/2/group/6/create/16. Information of the OneFlorida+ data is available at: https://onefloridaconsortium.org/. Request of INSIGHT data can be sent via: https://onefloridaconsortium.org/front-door/prep-to-research-data- query/.
Data analysis	For reproducibility, our codes are available at https://github.com/changsu10/Parkinson-Progression- Subtyping/tree/main. We used Python 3.7, python package pandas-1.3.5, numpy-1.19.5, scikit-learn-0.24.1, and pytorch-1.13.1 for machine learning and deep learning modeling. Statistical analyses were conducted using Python 3.7, python package scipy-1.2.1, statsmodels-0.11.1, and R 4.1.

For manuscripts utilizing custom algorithms or software that are central to the research but not yet described in published literature, software must be made available to editors and reviewers. We strongly encourage code deposition in a community repository (e.g. GitHub). See the Nature Portfolio guidelines for submitting code & software for further information.

Data

Policy information about availability of data

All manuscripts must include a data availability statement. This statement should provide the following information, where applicable:

- Accession codes, unique identifiers, or web links for publicly available datasets
- A description of any restrictions on data availability
- For clinical datasets or third party data, please ensure that the statement adheres to our policy

Data from the PPMI can be download from the official web site (http://www.ppmi-info.org) via request. Data from the PDBP can be download from AMP-PD platform (http://amp-pd.org) via request. Information of the INSIGHT database is available at https://insightcrn.org. Request of INSIGHT data can be sent via: https://nyc-cdrn.atlassian.net/servicedesk/customer/portal/2/group/6/create/16. Information of the OneFlorida+ data is available at: https://onefloridaconsortium.org/. Request of OneFlorida+ data can be sent via: https://onefloridaconsortium.org/front-door/prep-to-research-data-query/.

Research involving human participants, their data, or biological material

Policy information about studies with human participants or human data. See also policy information about sex, gender (identity/presentation), and sexual orientation and race, ethnicity and racism.

Reporting on sex and gender

Summary statistics on sex distributions of the PPMI and PDBP cohorts were reported in Supplementary Table 1. Summary statistics on sex distributions across the identified subtypes were reported in Table 1 and Supplementary Table 5.

From the INSIGHT database, we included 26139 male and 18565 female patients, who diagnosed with PD.

From the OneFlorida+ database, we included 42687 male and 36238 female patients, who diagnosed with PD.

Reporting on race, ethnicity, or other socially relevant groupings

Summary statistics on race distributions of the PPMI and PDBP cohorts were reported in Supplementary Table 1. Summary statistics on race distributions across the identified subtypes were reported in Table 1 and Supplementary Table 5.

In the INSIGHT database, we included 22834 white, 2790 black or African American, 2040 Asian, 15756 other race, and 1284 patients with unknown race information.

In the OneFlorida+ database, we included 53273 white, 7035 black or African American, 826 Asian, 103 American Indian or Alaska Native, 21 Native Hawaiian or Other Pacific Islander, 650 multiple race, 132 patients who refused to provide race information, 233 patients with no race information, 12324 other race, 4331 patients with unknown race information.

Population characteristics

For the PPMI and PDBP cohorts, details of the population characteristics including age, gender, primary diagnosis, and other PD-related variables (e.g., symptom duration, family history, education level) were described in the supplemental table 1.

The INSIGHT brings together top academic medical centers located in New York City (NYC), including Albert Einstein School of Medicine/Montefiore Medical Center, Columbia University and Weill Cornell Medicine/New York-Presbyterian Hospital, Icahn School of Medicine/Mount Sinai Health System, and New York University School of Medicine/Langone Medical Center. It contains longitudinal clinical data of over 15 million patients in the NYC metropolitan area.

OneFlorida+ includes 12 healthcare organizations and contains longitudinal and linked patient-level data, covering 17 million patients in Florida, 2.1 million in Georgia (via Emory), and 1.1 million in Alabama (via UAB Medicine) since 2012.

Recruitment

This study is a retrospective analysis and we did not perform subject recruitment.

Ethics oversight

The PPMI study protocol was approved by the institutional review board of the University of Rochester (NY, USA), as well as from each PPMI participating site. Data were obtained from PPMI website (https://www.ppmi-info.org) under PPMI Data Use Agreement.

The study protocol for each PDBP site was approved by institutional review board of each participating site. Data were obtained via the Accelerating Medicines Partnership Parkinson's disease (AMP-PD) platform (http://amp-pd.org) under AMP-PD Data Use Agreement.

We used de-identified patient data from INSIGHT and OneFlorida+. The use of the INSIGHT data was approved by the Institutional Review Board (IRB) of Weill Cornell Medicine under protocol 21-07023759. The use of the OneFlorida+ approved by the IRB of University of Florida under protocol IRB202300639.

Note that full information on the approval of the study protocol must also be provided in the manuscript.

Field-specific reporting

Please select the one below that is the best fit for your research. If you are not sure, read the appropriate sections before making your selection.

☒ Life sciences ☐ Behavioural & social sciences ☐ Ecological, evolutionary & environmental sciences

For a reference copy of the document with all sections, see nature.com/documents/nr-reporting-summary-flat.pdf

Life sciences study design

All studies must disclose on these points even when the disclosure is negative.

Sample size	In PPMI, we included 406 de novo PD participants (PD diagnosis within the last 2 years and untreated at enrollment), 188 healthy controls (HCs), and 61 participants who had dopamine transporter scans without evidence of dopaminergic deficit (SWEDD).<br><br>In PDBP, we included 210 early PD participants (symptom duration < 3 years at enrollment), 211 HCs, and 287 other PDs (who had a symptom duration > 3 years at enrollment).<br><br>For real-world data analysis for drug treatment effect estimation, we performed computational trial emulation to estimate treatment effects for drugs in the INSIGHT and OneFlorida+ data. The numbers of patients for each analysis using different subgroups are:<br>(1) Entire PD population: INSIGHT, n = 44704; OneFlorida+, n = 78928.<br>(2) Probable PD-R subtype population: INSIGHT, n = 10279; OneFlorida+, n = 26789.<br>(3) Statistics regarding eligible PD patients who received each tested drug in the INSIGHT and OneFlorida+ data were detailed in Figure 5.
Data exclusions	In the PPMI and PDBP cohorts, we excluded individuals who don't have follow-up information.<br><br>In the INSIGHT and OneFlorida+, we excluded patients whose ages were <50 at the first PD diagnosis event and patients who had neurodegenerative disease diagnoses before his/her first PD diagnosis
Replication	We originally derive PD subtypes using the PPMI data, and re-identified the subtypes in the PDBP cohort for validation.<br><br>For real-world data analysis for drug treatment effect estimation, we conducted analyses to obtain real-world evidence from two independent large scale databases, INSIGHT and OneFlorida+.
Randomization	This study is a retrospective analysis. We used de-identified individual-level data from research cohorts PPMI and PDBP, and real-world databases INSIGHT and OneFlorida+. Randomization is not applicable.
Blinding	This study is a retrospective analysis. We used de-identified individual-level data from research cohorts PPMI and PDBP, and real-world databases INSIGHT and OneFlorida+. Blinding is not applicable.

Reporting for specific materials, systems and methods

We require information from authors about some types of materials, experimental systems and methods used in many studies. Here, indicate whether each material, system or method listed is relevant to your study. If you are not sure if a list item applies to your research, read the appropriate section before selecting a response.

Materials & experimental systems

n/a	Involved in the study	
☒	☐	Antibodies
☒	☐	Eukaryotic cell lines
☒	☐	Palaeontology and archaeology
☒	☐	Animals and other organisms
☐	☒	Clinical data
☒	☐	Dual use research of concern
☒	☐	Plants

Methods

n/a	Involved in the study	
☒	☐	ChIP-seq
☒	☐	Flow cytometry
☒	☐	MRI-based neuroimaging

Clinical data

Policy information about clinical studies

All manuscripts should comply with the ICMJE guidelines for publication of clinical research and a completed CONSORT checklist must be included with all submissions.

Clinical trial registration	N/A, not a clinical trial
Study protocol	N/A, not a clinical trial
Data collection	For PD subtype identification, we used the longitudinal clinical data in the PPMI and PDBP cohorts.<br><br>We used Cerebrospinal Fluid biomarker data, neuroimaging data, genetic data, and gene expression data in the PPMI cohort for subtype-specific biomarker and molecular component identification.<br><br>For in silico drug repurposing, we transcriptomics-based drug-gene signature data in human cell lines from the Connectivity Map (Cmap) database.

	For drug treatment effect estimation, we used two large-scale real-world patient-level databases, INSIGHT and OneFlorida+.
Outcomes	Parkinson's disease, motor dysfunctions, cognitive decline, dementia, mental health problems, REM sleep disorder, etc.

Plants

Seed stocks	N/A
Novel plant genotypes	N/A
Authentication	N/A
